# Diabetic Distal Symmetrical Polyneuropathy: Correlation of Clinical, Laboratory, and Electrophysiologic Studies in Patients with Type 2 Diabetes Mellitus

**DOI:** 10.1155/2020/6356459

**Published:** 2020-07-03

**Authors:** Yi-Ching Weng, Sung-Sheng Tsai, Rong-Kuo Lyu, Chun-Che Chu, Long-Sun Ro, Ming-Feng Liao, Hong-Shiu Chang, Chiung-Mei Chen, Jawl-Shan Hwang, Hung-Chou Kuo

**Affiliations:** ^1^Department of Neurology, Chang Gung Memorial Hospital at Linkou Medical Center and Chang Gung University College of Medicine, Taoyuan, Taiwan; ^2^Department of Endocrinology, Chang Gung Memorial Hospital at Linkou Medical Center and Chang Gung University College of Medicine, Taoyuan, Taiwan; ^3^Chang Gung Memorial Hospital at Linkou Medical Center and Chang Gung University College of Medicine, Taoyuan, Taiwan

## Abstract

This cross-sectional study is aimed at determining the prevalence of distal symmetrical polyneuropathy (DSPN) and diabetic peripheral neuropathic pain (DPNP) in participants with type 2 diabetes mellitus (T2DM); finding the risk factors for DSPN and DPNP via biochemical tests; and correlating DSPN and DPNP with the results of electrophysiologic studies, quantitative sensory tests, and neurologic examination. The 145 participants with T2DM enrolled were divided into the DSPN (abnormal nerve conduction studies (NCS) with signs of polyneuropathy), subclinical DSPN (abnormal NCS without signs of polyneuropathy), minimal DSPN (normal NCS with signs of polyneuropathy), and no DSPN groups. The biochemical risk factors of diabetic peripheral neuropathy were investigated. Neurologic examinations, laboratory tests, NCS, vibration threshold tests, and thermal threshold tests were conducted. The modified Michigan Neuropathy Screening Instrument (mMNSI) and Douleur Neuropathique 4 were used to evaluate the severity of DSPN and DPNP, respectively. In all, 30% of participants had DSPN and 11% had DPNP. DSPN correlated strongly with male gender and higher glycohaemoglobin levels; NCS abnormality correlated with higher glycohaemoglobin levels; DSPN severity correlated with NCS of each stimulating nerve. DPNP commonly occurred with clinical and electrophysiologic evidence of DSPN. Symptomatic diabetic polyneuropathy significantly correlated with longer disease duration, higher glycohaemoglobin levels, and abnormal vibration tests. The thermal threshold test combined with nerve conduction tests could detect most of the patients with DSPN, subclinical DSPN, and minimal DSPN. Poor diabetic control was independently associated with the development of DSPN. DPNP was associated with DSPN. The combination of thermal threshold tests with NCS can potentially provide the diagnosis of DSPN.

## 1. Introduction

The complications of diabetes mellitus (DM) occur in multiple organs and systems, the most well-known complications being retinopathy, nephropathy, and neuropathy. Diabetic peripheral neuropathy occurs in 5-60% of persons with diabetes [1, 2]. Diagnosis is not easy as the presentations are broad, and almost half of diabetic peripheral neuropathies are symptom-free [3–5]. The most commonly occurring peripheral neuropathy in persons with diabetes is distal symmetrical polyneuropathy (DSPN) [6, 7]. Both the myelinated and unmyelinated nerve fibres may be influenced during the course of this condition. When myelinated nerve fibres are injured, persons present with numbness of the distal limbs, unsteady gait, and sometimes muscle atrophy and weakness. If unmyelinated fibres are influenced, painful neuropathy and autonomic symptoms may occur. Diabetic peripheral neuropathic pain (DPNP) affects around 26% of persons with diabetes and is present in approximately one-third of DSPN patients [7], impairing their ability to perform activities of daily living and affecting their mental quality of life [8]. With the goal of improving the diagnosis and screening of DSPN in patients with type 2 DM, new tests have been developed, and risk factors have been investigated in recent years [9–11]. However, the correlation of DM and diabetic peripheral neuropathy is complicated, and diagnosis standards and risk factors vary between studies. We conducted this study with the aim to demonstrate the application of a modified neuropathy screening instrument and electrophysiologic evaluation in screening patients with DSPN and analyse the relationship of demographic, clinical, and electrophysiologic variables in persons with type 2 DM with diabetic neuropathy and diabetic neuropathologic pain.

## 2. Materials and Methods

### 2.1. Subjects

Type 2 DM participants were enrolled in this study from August 2016 to July 2017 from the neurology and endocrinology outpatient departments of Chang Gung Memorial Hospital (CGMH), Linkou Medical Center, in Taiwan. Type 2 DM was diagnosed according to established diagnostic criteria, which include persons with insulin resistance with insulin secretion deficiency and (1) fasting plasma glucose ≥ 126 mg/dl (7.0 mmol/l) or (2) symptoms (such as polyuria, polydipsia, and unexplained weight loss) and a random plasma glucose ≥ 200 mg/dl (11.1 mmol/l) or (3) plasma glucose ≥ 200 mg/dl (11.1 mmol/l) 2 hours after a 75 g glucose load or (4) glycohaemoglobin (HbA1C) ≥ 48 mmol/mol (6.5%) [12].

Participants with type 2 DM who met all of the following criteria were enrolled in the study: (1) estimated glomerular filtration rate (eGFR) > 60 (ml/min/1.73 m^2^); (2) HbA1C ≤ 10 for at least 3 months prior to study inclusion (poor sugar control could lead to atypical painful neuropathy caused by glucose variability such as insulin neuritis; thus, HbA1C < 10 for a longer duration is preferred to minimize patients with atypical painful neuropathy); (3) body mass index (BMI) of 20-35; and (4) medical history of type 2 DM < 20 years. Potential participants who met any of the following exclusion criteria were not allowed to enter the study: (1) history of major cardiac, cerebral, or peripheral vascular diseases or congestive heart failure; (2) liver cirrhosis or malignancies in the past year; (3) certain forms of neuropathy (other than diabetic neuropathy) which occur more frequently in those with diabetes than in the general population (including chronic inflammatory demyelinating polyneuropathy, neuropathy due to vitamin B_12_ deficiency, hypothyroidism, autoimmune disease, paraproteinaemia, and uraemia); (4) recent history of exposure to a neurotoxic agent or heavy metals; (5) family history of hereditary peripheral neuropathy; or (6) abuse of illicit drugs or alcoholism within 1 year.

All participants in this study gave written informed consent, and the protocol was approved by the Institutional Review Board of CGMH (104-9521B, 105-5438C, and 201700994B0). The study conformed to the Declaration of Helsinki, US Federal Policy for the Protection of Human Subjects, or European Medicines Agency Guidelines for Good Clinical Practice. All participants received complete neurological examinations and electrophysiologic studies, and their fasting blood was drawn. All participants received blood tests to rule out other systemic diseases and evaluate for possible risk factors, including whole blood counts, erythrocyte sedimentation rate, fasting plasma sugar levels, HbA1C, cholesterol, triglycerides, renal and liver function, serum vitamin B_12_, homocysteine, and folic acid levels. Serological tests for syphilis, autoimmune disease (antinuclear antibody test, rheumatoid factor, and anti-Sjögren's syndrome-related antigen A/B), and endocrine function of both the thyroid and adrenal glands were also performed. Serum protein electrophoresis, immunofixation electrophoresis, and cryoglobulin tests were also performed.

### 2.2. Evaluation of Distal Symmetrical Polyneuropathy (DSPN)

An instrument modified in 2000 from the Michigan Neuropathy Screening Instrument (MNSI), the modified Michigan Neuropathy Screening Instrument (mMNSI), was used to assess neuropathy in participants [13]. Instead of performing vibration sensation tests and checking ankle reflexes by physical inspection (as performed in the MNSI), assessments of vibration perception thresholds and thermal perception thresholds were done using NeuroSensory analysers which quantitatively evaluate large and small fibre dysfunction in this study. Measurement using NeuroSensory analysers is quantitative, objective, and precise, compared to the monofilament and tuning fork tests used in the MNSI physical examination. The mMNSI is a clinical and semiquantified evaluation of neuropathy that includes foot appearance (0 and 1 for normal and abnormal, respectively), ulceration (0 and 1 for normal and abnormal, respectively), ankle reflex (0, 0.5, and 1 for normal, reenforced, and absent, respectively), thermal test (0 and 1 for normal and abnormal, respectively), and vibration test (0 and 1 for normal and abnormal, respectively) of feet on both sides. The mMNSI score ranges between 0 and 10. In the mMNSI, we used a quantitative thermal threshold test (Medoc Thermal Sensory Analyser (TSA) 2001 devices, Medoc Ltd. Advanced Medical Systems, Ramat Yishai, Israel) to replace the monofilament examination and a quantitative vibration threshold test (VSA-3000, Medoc Ltd. Advanced Medical Systems, Ramat Yishai, Israel) to replace the tuning fork vibration test of the MNSI (Table S1).

Nerve conduction studies (NCS) were performed by conventional supramaximal percutaneous stimulation of the limbs and surface recordings according to the Toronto consensus recommendation for diagnosing diabetic peripheral neuropathies [14]. Briefly, five different nerve studies were conducted on each side, including the ulnar, tibial, and peroneal motor conduction velocity; the ulnar sensory conduction velocity; and the sural amplitude or conduction velocity (Table S2). Results were defined as abnormal if two or more nerves on each side were abnormal and had to include at least one nerve in the lower limb, compared with age-matched controls in our laboratory.

### 2.3. Definition of DSPN and Its Severity

We classified participants into groups with DSPN, subclinical DSPN, minimal DSPN, and no DSPN. DSPN has been defined in the literature as abnormal nerve conduction combined with symptoms or signs of polyneuropathy; subclinical DSPN is defined as abnormal nerve conduction studies (NCS) without symptoms or signs of polyneuropathy [14]. Minimal DSPN, a term we define in this study which has not been used previously, was normal NCS with signs of polyneuropathy. In our study, DSPN was defined as abnormal NCS with a total score ≥ 2.5 of 10 on the mMNSI questionnaire; subclinical DSPN was defined as abnormal NCS with a total mMNSI score < 2.5 and abnormal NCS; minimal DSPN was defined as normal NCS (less than 2 of 5 nerves abnormal on NCS, with a total mMNSI score ≥ 2.5). The severity of DSPN, according to NCS results, was classified as mild if 2 nerves were abnormal, moderate if 3-4 nerves were abnormal, and severe if all 5 nerves were abnormal. Symptomatic DSPN was defined as complaint of symmetrical, bilateral, distal sensory and/or motor defects.

### 2.4. Evaluation of Diabetic Peripheral Neuropathic Pain

The Douleur Neuropathique 4 (DN4) questionnaire has been validated for use in diabetic patients [15]. The Taiwan version of the DN4 (DN4-T) score was used to evaluate DPNP in our study [16]. The DN4 consist of a series of four groups of questions consisting of seven sensory descriptors (burning, painful cold, electric shock, tingling, pins and needles, numbness, and itching) and three signs related to a sensory physical examination of the painful area (tactile hypaesthesia, pinprick hypaesthesia, and allodynia) [17]. DPNP in this study was defined as a total DN4-T score of 4 or more out of 10 in both lower legs, excluding those with neuralgia noted only in the hand or related carpal tunnel syndrome.

### 2.5. Statistical Analysis

The distribution of mMNSI total score and DPNP rate were summarized as mean ± standard deviation (SD) and *n* (%) by DSPN stage, respectively; and compared with no DSPN, each DSPN stage was analysed using the two-sample *t*-test and Pearson chi-squared test. Demographics, clinical characteristics, electrophysiologic studies, and neurologic examination were presented as mean ± SD for continuous data and *n* (%) for categorical data by group. Differences between two groups or comparisons of DSPN stage with no DSPN were conducted using the two-sample *t*-test for continuous data and Pearson chi-squared test for categorical ones. Furthermore, the differences in demographics and clinical characteristics by DSPN severity (mild, moderate, and severe) were also compared using the one-way ANOVA test for continuous variables and the chi-squared test for categorical ones. All statistical assessments were two-tailed and considered significant at *p* < 0.05. Statistical analyses were performed and derived from Microsoft Office Excel and http://vassarstats.net/odds2x2.html.

## 3. Results

### 3.1. Demographic and Biochemical Results

**Figure 1:**
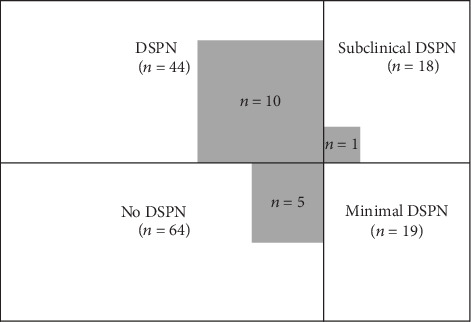
The distribution of type 2 DM patients with or without DSPN. DM: diabetes mellitus; DSPN: distal symmetrical polyneuropathy; DPNP: diabetic peripheral neuropathic pain; mMNSI: modified Michigan Neuropathy Screening Instrument; NCS: nerve conduction studies. No DSPN: type 2 DM with mMNSI < 2.5 and NCS severity score = 0-1. Minimal DSPN: type 2 DM with mMNSI ≥ 2.5 and NCS severity score = 0-1. Subclinical DSPN: type 2 DM with mMNSI < 2.5 and NCS severity score ≥ 2. DSPN: type 2 DM with mMNSI ≥ 2.5 and NCS severity score ≥ 2. Grey: DPNP including in each subgroup, type 2 DM with DN4 ≥ 4.

One hundred forty-five participants with type 2 DM were enrolled in this study, including 54 women and 91 men. In all, 82 participants had mMNSI < 2.5 and 63 had mMNSI ≥ 2.5. Sixty-four participants had no DSPN (44%, women/men: 34/30), 19 had minimal DSPN (13%, women/men: 6/13), 18 had subclinical DSPN (12%, women/men: 7/11), and 44 had DSPN (30%, women/men: 7/37, *p* ≤ 0.0001). The number of patients in each group is summarized in Figure 1, and the distribution of mMNSI total scores is summarized in Table 1. The mean age at enrolment was 58.2, 58.5, 58.6, and 54.6 years for the no DSPN, minimal DSPN, subclinical DSPN, and DSPN groups, respectively. The mean disease duration was 6.5 years in the no DSPN group, 7.9 years in the minimal DSPN group, 6.8 years in the subclinical DSPN group, and 7.5 years in the DSPN group. HbA1C levels were 53, 51, 66, and 65 mmol/mol (7.0, 6.8, 8.2, and 8.1%) for the no DSPN, minimal DSPN, subclinical DSPN, and DSNP groups, respectively. The HbA1C levels were statistically significantly higher in the groups with abnormal NCS (DSPN and subclinical DSPN groups) (*p* < 0.0001) compared to the no DSPN group. There were no obvious statistically significant differences in disease duration, hypertension, total cholesterol level, high- and low-density lipoprotein cholesterol, triglyceride level, eGFR, and BMI between groups. In the DSPN subgroups, the severity of DSPN was not correlated with any of the demographic data or laboratory tests. All demographic data and laboratory results are listed in Table 2.

### 3.2. Electrophysiological Studies and Physical Assessment

For the 44 DSPN participants, 18 had mild DSPN, 14 had moderate DSPN, and 12 had severe DSPN. Abnormal thermal tests occurred much more frequently in both the minimal DSPN and severe DSPN participants when compared to the no DSPN participants (*p* < 0.0001). Abnormal vibration threshold tests showed statistically significant differences in the DSPN and minimal DSPN groups (*p* < 0.0001) when compared to the no DSPN group. Almost all participants with minimal DSPN and DSPN had reduced ankle reflexes. When compared to participants without DSPN, DSPN participants tended to be more symptomatic and have more DPNP (*p* < 0.0001 and *p* < 0.05, respectively). In the DSPN subgroups, the severity of DSPN was not correlated with abnormalities in the physical assessment, bilateral focal median neuropathy at the wrist on NCS, and thermal or vibration tests (Table 3). The combination of NCS and thermal threshold tests could identify most of the patients with DSPN and minimal DSPN. However, in the subclinical DSPN group, the incidence of abnormal thermal or vibration tests was low (Table 3). The DSPN, subclinical DSPN, and DPNP participants showed statistically significant differences in NCS in almost all parameters of sensory and motor nerves when compared to the no DSPN participants. Three degrees of DSPN severity also showed correlation with the NCS results of all tested nerves (Table S3).

### 3.3. Diabetic Peripheral Neuropathic Pain and Symptomatic Polyneuropathy

In total, 16 DPNP patients were found to have no DSPN, subclinical DSPN, or DSPN (5, 1, and 10 subjects, respectively), which accounted for 11% of all participants. DPNP was not correlated with any demographic and laboratory parameters. DPNP patients showed statistically significant differences in NCS, mMNSI, and NCS scores of neuropathy when compared with no DPNP patients; however, no significant differences were found for thermal or vibration threshold tests (Table 4).

When participants were divided by whether or not they were symptomatic, symptomatic participants were found to have longer disease duration, higher HbA1C, and higher total cholesterol (*p* < 0.0001, *p* < 0.0001, and *p* < 0.05, respectively). Also, symptomatic participants had more reduced ankle reflexes, bilateral focal median neuropathy at the wrist on NCS, and abnormal thermal and vibration threshold tests when compared with asymptomatic patients (*p* < 0.0001, *p* < 0.05, *p* < 0.05, and *p* < 0.0001, respectively) (Table 5).

### 3.4. Focal Median Neuropathies at the Wrist

The NCS evidence of focal median neuropathy at the wrist occurred in 56% of participants. At the same time, only 12% of participants had clinical experience of carpal tunnel syndrome. For clinical and NCS evidence of DSPN, 51 of 81 participants (63%) had bilateral focal median neuropathy at the wrist on NCS. For DPNP patients, 2 of 5 subjects with no DSPN (40%) had NCS evidence of focal median neuropathy, but only 1 of 10 patients with DSPN (10%) had focal median neuropathy (data not shown). Results of focal median neuropathy at the wrist are summarized in Table S4.

## 4. Discussion

In this study, the risk factors identified for screening DSPN included longer disease duration, higher glycohaemoglobin levels, and abnormal vibration and thermal threshold tests. The combination of thermal threshold tests and NCS could indicate a diagnosis of DSPN. Further, DSPN as defined in our study using modified evaluation methods was correlated with DPNP and symptomatic neuropathy. Our preliminary results in using the mMNSI suggest that it could be used to screen patients with painful or symptomatic neuropathy, to improve patient care.

The clinical presentation of DSPN is known to be broad. Methods of diagnosis include using a screening instrument and conducting electrophysiologic evaluations. A previous study has shown that use of MNSI alone often cannot detect lower levels of diabetic peripheral neuropathy, and combined use of multiple methods is recommended to obtain a diagnosis [18–20]. The Toronto consensus recommended the use of abnormal NCS with a symptom or sign to diagnose diabetic peripheral neuropathy [14]. Although NCS provides an objective means of quantifying peripheral large nerve fibre dysfunction, it cannot assess small sensory fibre damage, one of the earliest manifestations of diabetic peripheral neuropathy. A study in DM patients with/without sensory symptoms and normal NCS showed that intraepidermal nerve fibre density and thermal thresholds were significantly reduced compared to those in healthy controls; in addition, they also differed between the symptomatic and asymptomatic groups [21].

Using our modified evaluation method, we defined a group of patients as having “minimal DSPN” who had normal NCS results but signs of DSPN (altered vibration or thermal thresholds). Our modified version of the MNSI is more objective in screening patients with DSPN and can identify small sensory fibre damage, one of the earliest manifestations of DSPN.

Our results demonstrated that a modified semiquantitative vibration thermal threshold test combined with nerve conduction tests could identify most of the patients with DSPN, subclinical DSPN, and minimal DSPN. Further, our results indicate that our evaluation method could easily differentiate between patients with DSPN and no DSPN with only a partial NCS. There is no need to test all five nerves; mMNSI plus a positive result for any one of the five nerves could screen for DSPN. This makes the test clinically more efficient and easier for patients to bear.

The mechanisms of diabetic peripheral neuropathy are complicated. Glucose and lipids are involved in the pathway and subsequent osmotic stress, electron transport overload, and formation of reactive oxygen species, with the result of DNA damage and cell apoptosis [22]. In a previous study, newly diagnosed persons with type 2 DM had a high prevalence of diabetic neuropathy, as much as 60% when measured by NCS and 39% when measured by a vibration threshold test [23]. The abnormality of each electrophysiologic test varies between studies due to underlying differences between populations. The abnormal rates were 25-63% for NCS [24–28], 39-63% for vibration threshold tests [23, 29], and 22-81% for thermal threshold tests [21, 29, 30]. Intraepidermal nerve fibre (IENF) density showed 25-81% abnormality [21, 29]. In our study, NCS were abnormal in 42% of participants, thermal tests abnormal in 42%, and vibration tests abnormal in 20%. Some studies have suggested comparable results between quantitative sensory tests and NCS, indicating the possible use of the latter rather than the former to evaluate diabetic neuropathy [31, 32]. However, NCS combined with thermal threshold tests could detect most of those with DSPN and minimal DSPN in our study. Moreover, if DM patients have no obvious foot appearance abnormality or decreased ankle tendon reflexes, NCS are the most practical way to detect patients with subclinical DSPN rather than thermal or vibration tests, according to our results.

The electrophysiologic results are associated with the HbA1C level, disease duration, and participant age [26, 30, 33–36]. In one nerve conduction study, HbA1C above 75 mmol/mol (9%) suggested greater sensory nerve abnormality [34]. In another study, HbA1C above 69 mmol/mol (8.5%) resulted in a greater change in the conduction velocities of motor nerves in the lower limbs during a 2-year follow-up period [33]. Moreover, after treatment for diabetes, the diabetic neuropathy as assessed by NCS may improve [37]. Although not all studies show the importance of hyperglycaemia control, the obvious conclusion is that good glucose control can retard the deterioration of the peripheral nerves or even improve conditions in the early stages of disease. In a recent study, HbA1C variability was a risk factor for the development of diabetic neuropathy [38]. In one large cohort study, 62% of asymptomatic participants had abnormal nerve conduction results if their disease duration was more than 15 years, but only 25% had abnormal results if their disease duration was less than 15 years [26]. This result emphasizes the importance of disease duration in diabetic neuropathy, which is the effect of long-term hyperglycaemia. Small fibre tests like the thermal threshold test have also been found to be correlated with the participants' age, disease duration, and HbA1C level [30]. IENF density was negatively associated with disease duration [29]. Certain parameters may relate to diabetic neuropathy, including height, diabetic nephropathy, diabetic retinopathy, hypertension, low high-density lipoprotein cholesterol levels, smoking, obesity, and hypertriglyceridaemia [26, 33, 39, 40]. The higher HbA1C level and male gender correlated with DSPN, and the higher HbA1C level was also correlated with NCS abnormalities, but not DSPN severity, in our study. Vascular risk factors investigated in our study were not correlated with DSPN.

Diabetic neuropathy symptoms serve as sentinels for clinical morbidity, and nerve conduction results are more likely to be abnormal in symptomatic participants [27, 35]. IENF density is reduced in both the symptomatic and asymptomatic persons, but the reduction is more obvious in symptomatic persons [21]. Symptomatic persons are older and have more abnormal thermal tests, as well as longer disease duration [30]. Old age, longer disease duration, greater HbA1C variability, higher HbA1C values, and the presence of hypertension were also found to predict symptomatic neuropathy [38]. Participants with painful neuropathy and those with foot ulcers had poorer autonomic and nerve conduction study results compared to those with nonpainful neuropathy [41]. Cold thermal tests, vibration tests, and the absence of deep tendon reflex are all correlated with painful neuropathy [42]. In our study, symptomatic DSPN participants were more likely to have abnormal vibration tests, longer disease duration, high levels of HbA1C, and decreased ankle reflex (*p* ≤ 0.0001). Higher total cholesterol levels and abnormal thermal tests were also correlated with symptomatic neuropathy (*p* ≤ 0.05). Painful diabetic neuropathy was correlated with the severity and abnormality of NCS, but not the abnormal thermal test in our study. DPNP was also not correlated with any laboratory tests, including HbA1C levels. In clinical practice, asymptomatic patients may have abnormal electrophysiologic results and some symptomatic patients may have normal electrophysiologic results. This variability may be partially explained by individual threshold differences and the slow progression of DM that make symptoms tolerable. The latter may also relate to the relative sensitivity of each electrophysiologic examination.

Previous studies had debated which nerve is most useful for detecting diabetic neuropathy. Nerves of the lower limb such as the sural nerve would be more sensitive because diabetic neuropathy follows a length-dependent pattern, and sensory nerves would be more sensitive than motor nerves [43, 44]. The machines used for pure sural nerve examination such as “DPN-Check” have been used to screen for diabetic neuropathy [45]. The interdigital nerve, dorsal sural nerve, medial plantar nerve, and a combination of the dorsal sural and medial plantar nerves have all been proposed as candidate nerves for better detection of diabetic neuropathy rather than the traditional sural or superficial peroneal nerves, because these two nerves are not distal enough [46–51]. However, due to the technical difficulty related to those distal nerves, they cannot be used broadly in clinical practice [44]. In our study, all the nerves, including the ulnar, sural, deep peroneal, and tibial nerves, showed significant differences between the DSPN and no DSPN groups, together with differences in the minimal F wave. This result could be because of the original study design. In our view, traditional NCS have good discrimination ability between patients with and without DSPN.

Newly diagnosed persons with diabetes have more abnormal results in NCS in the upper limbs than in the lower limbs [23]. We also found a clinical history of carpal tunnel syndrome in participants, although the NCS evidence of bilateral focal median neuropathy at the wrist was still very frequent. DSPN is the most common type of diabetic neuropathy, but focal/multifocal neuropathies can occur in persons with diabetes [6, 52, 53]. Many studies report rates of asymptomatic median neuropathy of 6-63% in participants with diabetes [6, 25, 54–56]. In our study, 51% of participants had bilateral median neuropathy at the wrist. Median neuropathy at the wrist is a presentation of subclinical focal diabetic neuropathy in such patients [57, 58] and is a manifestation of increased vulnerability in terms of entrapment [25, 52, 54]. Although the incidence rate of symptomatic carpal tunnel syndrome in persons with diabetes does not differ from that of those without diabetes [59], those with diabetes do have an increased lifetime risk of carpal tunnel syndrome [52].

Males are more prone than females to diabetic peripheral neuropathy, a result already mentioned in other studies of persons with both type 1 and type 2 DM [34, 60]. We similarly found more significant neuropathic changes in male participants than in females. The underlying mechanism remains uncertain, but a genetic component influencing diabetic neuropathy has been proposed [60].

One of the limitations of this study is that we did not have the result from using a standard MNSI questionnaire; thus, we were not able to calculate sensitivity, specificity, and negative and positive predictive values of the mMNSI for the presence or diagnosis of DSPN and DPNP in relation to the standard MNSI. Some studies use NCS as a gold standard for diagnosing DSPN, but as we wanted to demonstrate that patients with normal NCS could have signs of early DSPN (the minimal DSPN group), we did not use NCS as a gold standard for diagnosis. However, from the current results, we can preliminarily demonstrate that, in patients with abnormal vibration or thermal tests, we can identify a proportion of those with minimal DSPN. We also showed that patients with mMNSI > 2.5 and abnormal vibration or thermal test results are likely to have DSPN (minimal DSPN); and patients with mMNSI < 2.5 but with normal vibration and thermal test results are likely to have no DSPN (Table S5). Our subsequent study will include an appropriate “gold standard,” in order to compare the effectiveness of the mMNSI, in combination with different electrophysiologic evaluations, to confirm DSPN.

In conclusion, diabetic neuropathy is common in persons with diabetes. For type 2 DM, poor blood sugar control remains the strongest risk factor for DSPN. The combined use of NCS with thermal threshold tests could allow for screening of patients with possible DSPN. DPNP was more prone to occur in patients with DSPN. These results could help clinicians refine their practice to reduce morbidity and mortality in persons with diabetes.

## Figures and Tables

**Table 1:** Distribution of mMNSI total score and DPNP^a^ rate by DSPN stage^b^.

	Total	No DSPN	Minimal DSPN	Subclinical DSPN	DSPN
*Patient no.*	*145*	*64*	*19*	*18*	*44*
mMNSI, mean ± SD total score	2.7 ± 0.7	1 ± 1	4 ± 0^∗∗∗^	2 ± 1	5 ± 1^∗∗∗^
DPNP, *n* (%)	16 (11.0)	5 (7.8)	0 (0.0)	1 (5.6)	10 (22.7)^∗^

Abbreviations: mMNSI: modified Michigan Neuropathy Screening Instrument; DSPN: distal symmetrical polyneuropathy; DPNP: diabetic peripheral neuropathic pain; NCS: nerve conduction studies; DN4: Douleur Neuropathique; SD: standard deviations. ^a^DPNP indicates patients with DN4≧4. ^b^No DSPN, patients with mMNSI total score < 2.5 and NCS severity score 0-1; minimal DSPN, patients with mMNSI total score≧2.5 and NCS severity score 0-1; subclinical DSPN, patients with mMNSI total score < 2.5 and NCS severity score≧2; DSPN, patients with mMNSI total score≧2.5 and NCS severity score≧2. Data were compared by DSPN stage against no DSPN, using the two-sample *t*-test for the mMNSI total score and the chi-squared test for the DPNP rate. ^∗^*p* < 0.05, ^∗∗^*p* < 0.01, and ^∗∗∗^*p* < 0.0001 indicate significant difference as compared with no DSPN.

**Table 2:** Demographics and clinical characteristics by DSPN stage^a^.

Variables	No DSPN	Minimal DSPN	Subclinical DSPN	DSPN			
				DSPN	Mild DSPN	Moderate DSPN	Severe DSPN
*Patient no.*	*64*	*19*	*18*	*44*	*18*	*14*	*12*
Gender (F/M)	34/30	6/13	7/11	7/37^∗∗∗^	3/15	4/10	0/12
Age (y)	58.2 ± 7.1	58.5 ± 8.6	58.6 ± 7.8	54.6 ± 10.0^∗^	57.7 ± 8.3	50.1 ± 12.4	55.2 ± 7.9
BMI (kg/m^2^)	25.7 ± 3.8	26.4 ± 3.4	25.5 ± 4.5	25.9 ± 4.1	26.0 ± 3.5	26.1 ± 4.7	25.5 ± 4.6
Duration of T2DM (y)	6.5 ± 5.4	7.9 ± 5.4	6.8 ± 5.2	7.5 ± 5.5	8.6 ± 5.8	7.4 ± 4.6	6.9 ± 6.4
HTN, *n* (%)	4 (6.3)	0 (0.0)	1 (5.6)	2 (4.5)	2 (11.1)	0 (0.0)	0 (0.0)
HbA1C (%)	7.0 ± 0.8	6.8 ± 0.8	8.2 ± 2.0^∗∗∗^	8.1 ± 1.7^∗∗∗^	7.6 ± 1.0	8.8 ± 2.0	8.0 ± 1.9
T-CHOL (mg/dl)	166 ± 34	162 ± 30	162 ± 33	176 ± 35	177 ± 34	167 ± 27	185 ± 44
HDL-C (mg/dl)	47 ± 11	43 ± 9	48 ± 13	46 ± 14	43 ± 8	44 ± 15	54 ± 17
LDL-C (mg/dl)	91 ± 27	90 ± 24	82 ± 19	101 ± 30	111 ± 31	88 ± 32	101 ± 23
TG (mg/dl)	143 ± 85	148 ± 53	146 ± 125	138 ± 73	131 ± 60	181 ± 87	99 ± 49
Vit. B_12_ (pg/ml)	862 ± 813	831 ± 488	1037 ± 788	815 ± 581	639 ± 342	1068 ± 825	826 ± 523
Hom (*μ*mol/l)	10 ± 3	11 ± 3	9 ± 2	11 ± 3	11 ± 2	11 ± 4	11 ± 4
eGFR (ml/min/1.73m^2^)	106 ± 25	92 ± 23^∗^	111 ± 27	99 ± 27	90 ± 20	116 ± 31	93 ± 23

Abbreviations: mMNSI: modified Michigan Neuropathy Screening Instrument; DSPN: distal symmetrical polyneuropathy; DPNP: diabetic peripheral neuropathic pain; NCS: nerve conduction studies; DN4: Douleur Neuropathique; F: females; M: males; y: years; BMI: body mass index; T2DM: type II diabetes mellitus; HTN: hypertension; HbA1C: glycated haemoglobin; Vit. B_12_: vitamin B_12_; HTN: hypertension; Hom: homocysteine; T-CHOL: total cholesterol; HDL-C: high-density lipoprotein cholesterol; LDL-C: low-density lipoprotein cholesterol; TG: triglyceride; eGFR: estimated glomerular filtration rate. ^a^ No DSPN, patients with mMNSI total score < 2.5 and NCS severity score 0-1; minimal DSPN, patients with mMNSI total score≧2.5 and NCS severity score 0-1; subclinical DSPN, patients with mMNSI total score < 2.5 and NCS severity score≧2; DSPN, patients with mMNSI total score≧2.5 and NCS severity score≧2. Data were presented as *n* of F/M for gender, *n* (%) for categorical variables, and mean ± standard deviations (SD) for continuous values given DSPN stage. Data were compared with DSPN stage, no DSPN using the chi-squared test for categorical variables and the two-sample *t*-test for continuous ones. ^∗^*p* < 0.05, ^∗∗^*p* < 0.01, and ^∗∗∗^*p* < 0.0001 indicate significant difference as compared with no DSPN. Data were also compared among DSPN severity (mild, moderate, and severe DSPN) via the one-way ANOVA test for continuous variables and the chi-squared test for categorical variables; and no significance was derived among DSPN severity.

**Table 3:** Electrophysiologic studies and neurologic examinations of patients by DSPN stage^a^ with mMNSI < 2.5 or ≥2.5.

	No DSPN	Minimal DSPN	Subclinical DSPN	DSPN			
				All DSPN	Mild DSPN	Moderate DSPN	Severe DSPN
Total							
*Patient no.*	*64*	*19*	*18*	*44*	*18*	*14*	*12*
Symptoms of polyneuropathy, *n* (%)	12 (18.8)	4 (21.1)	11 (61.1)^∗∗∗^	32 (72.7)^∗∗∗^	12 (66.7)	11 (78.6)	9 (75.0)
Reduction of ankle reflex, *n* (%)	40 (62.5)	19 (100.0)^∗∗^	14 (77.8)	43 (97.7)^∗∗∗^	17 (94.4)	14 (100.0)	12 (100.0)
Bilateral median neuropathy at wrists, *n* (%)	26 (40.6)	8 (42.1)^∗^	13 (72.2)	27 (61.4)^∗^	10 (55.6)	11 (78.6)	6 (50.0)
Abnormal thermal test, *n* (%)	6 (9.4)	16 (84.2)^∗∗∗^	0 (0.0)	40 (90.9)^∗∗∗^	15 (83.3)	14 (100.0)	11 (91.7)
Abnormal vibration, *n* (%)	0 (0.0)	4 (21.1)^∗∗∗^	2 (11.1)	24 (54.5)^∗∗∗^	9 (50.0)	8 (57.1)	7 (58.3)
mMNSI < 2.5							
*Patient no.*	*64*	*0*	*18*	*0*	*0*	*0*	*0*
Symptoms of polyneuropathy, *n* (%)	12 (18.8)	—	11 (61.1)	—	—	—	—
Reduction of ankle reflex, *n* (%)	24 (37.5)	—	4 (22.2)	—	—	—	—
Bilateral median neuropathy at wrists, *n* (%)	26 (40.6)	—	13 (72.2)	—	—	—	—
Abnormal thermal test, *n* (%)	6 (9.4)	—	0 (0.0)	—	—	—	—
Abnormal vibration, *n* (%)	0 (0.0)	—	2 (11.1)	—	—	—	—
mMNSI ≥ 2.5							
*Patient no.*	*0*	*19*	*0*	*44*	*18*	*14*	*12*
Symptoms of polyneuropathy, *n* (%)	—	4 (21.1)	—	32 (72.7)	12 (66.7)	11 (78.6)	9 (75.0)
Reduction of ankle reflex, *n* (%)	—	0 (0.0)	—	43 (97.7)	17 (94.4)	14 (100)	12 (100)
Bilateral median neuropathy at wrists, *n* (%)	—	8 (42.1)	—	27 (61.4)	10 (55.6)	11 (78.6)	6 (50.0)
Abnormal thermal test, *n* (%)	—	16 (84.2)	—	40 (90.9)	15 (83.3)	14 (100.0)	11 (91.7)
Abnormal vibration, *n* (%)	—	4 (21.1)	—	24 (54.4)	9 (50.0)	8 (57.1)	7 (58.3)

Abbreviations: mMNSI: modified Michigan Neuropathy Screening Instrument; DSPN: distal symmetrical polyneuropathy; DPNP: diabetic peripheral neuropathic pain; NCS: nerve conduction studies. ^a^No DSPN, patients with mMNSI total score < 2.5 and NCS severity score 0-1; minimal DSPN, patients with mMNSI total score≧2.5 and NCS severity score 0-1; subclinical DSPN, patients with mMNSI total score < 2.5 and NCS severity score≧2; DSPN, patients with mMNSI total score≧2.5 and NCS severity score≧2. Data were compared with DSPN stage, no DSPN using the chi-squared test. ^∗^*p* < 0.05, ^∗∗^*p* < 0.01, and ^∗∗∗^*p* < 0.0001 indicate significant difference as compared with no DSPN.

**Table 4:** Demographic characteristics, neurologic examinations, and electrophysiologic studies of patients with or without DPNP^a^.

Variables	No DPNP	DPNP
*Patient no.*	*129*	*16*
Gender (F/M)	48/81	6/10
Age (y)	57.5 ± 8.1	54.6 ± 11.1
BMI (kg/m^2^)	25.9 ± 3.8	25.3 ± 5.1
Duration of T2DM (y)	7.0 ± 5.3	7.8 ± 6.3
HTN, *n* (%)	5 (3.9)	2 (12.5)
HbA1C (%)	7.4 ± 1.5	7.6 ± 0.9
T-CHOL (mg/dl)	168 ± 34	172 ± 32
HDL-C (mg/dl)	46 ± 12	46 ± 12
LDL-C (mg/dl)	92 ± 26	94 ± 36
TG (mg/dl)	140 ± 84	163 ± 78
Vit. B_12_ (pg/ml)	860 ± 731	914 ± 461
Hom (*μ*mol/l)	10 ± 3	11 ± 3
eGFR (ml/min/1.73m^2^)	102 ± 25	104 ± 31

NCS score of neuropathy	1 ± 2	2 ± 2^∗^
No. of abnormal NCS	51 (39.5)	11 (68.8)^∗^

mMNSI, total score	2 ± 2	4 ± 2^∗^
Symptoms of polyneuropathy	43 (33.3)	16 (100.0)^∗∗∗^
Reduction of ankle reflex	101 (78.3)	15 (93.8)
Bilateral median neuropathy at wrists	66 (51.2)	8 (50.0)
Abnormal thermal test	52 (40.3)	10 (62.5)
Abnormal vibration	24 (18.6)	6 (37.5)

Abbreviations: mMNSI: modified Michigan Neuropathy Screening Instrument; DSPN: distal symmetrical polyneuropathy; DPNP: diabetic peripheral neuropathic pain; NCS: nerve conduction studies; DN4: Douleur Neuropathique; F: females; M: males; y: years; BMI: body mass index; T2DM: type II diabetes mellitus; HTN: hypertension; HbA1C: glycated haemoglobin; Vit. B_12_: vitamin B_12_; HTN: hypertension; Hom: homocysteine; T-CHOL: total cholesterol; HDL-C: high-density lipoprotein cholesterol; LDL-C: low-density lipoprotein cholesterol; TG: triglyceride; eGFR: estimated glomerular filtration rate. ^a^DPNP indicates patients with DN4≧4. Data were presented as *n* of F/M for gender, *n* (%) for categorical variables, and mean ± standard deviations (SD) for continuous values by patients with and without DPNP. Data were compared using the chi-squared test for categorical variables and the two-sample *t*-test for continuous ones. ^∗^*p* < 0.05, ^∗∗^*p* < 0.01, and ^∗∗∗^*p* < 0.0001 indicate significant difference in comparing patients with and without DPNP.

**Table 5:** Demographic characteristics, neurologic examinations, and electrophysiologic studies for the symptomatic and asymptomatic^a^ patients.

Variables	Asymptomatic	Symptomatic
*Patient no.*	*86*	*59*
Gender (F/M)	33/53	21/38
Age (y)	58.1 ± 7.5	55.9 ± 9.6
BMI (kg/m^2^)	26.0 ± 3.9	25.6 ± 3.9
Duration of T2DM (y)	5.8 ± 4.8	9.1 ± 5.6^∗∗∗^
HTN, *n* (%)	4 (4.7)	3 (5.1)
HbA1C (%)	7.0 ± 1.0	8.1 ± 1.7^∗∗∗^
T-CHOL (mg/dl)	163 ± 34	176 ± 32^∗^
HDL-C (mg/dl)	45 ± 11	48 ± 14
LDL-C (mg/dl)	90 ± 28	96 ± 25
TG (mg/dl)	143 ± 79	141 ± 90
Vit. B_12_ (pg/ml)	776 ± 677	994 ± 728
Hom (*μ*mol/l)	10 ± 3	10 ± 3
eGFR (ml/min/1.73m^2^)	101 ± 24	105 ± 29
DPNP, *n* (%)	0 (0.0)	16 (27.1)^∗∗∗^

mMNSI, total score	2 ± 1	4 ± 2^∗∗∗^
Symptoms of polyneuropathy	0 (0)	59 (100)
Reduction of ankle reflex	59 (68.6)	57 (96.6)^∗∗∗^
Bilateral median neuropathy at wrists	36 (41.9)	38 (64.4)^∗∗^
Abnormal thermal test	30 (34.9)	32 (54.2)^∗^
Abnormal vibration	8 (9.3)	22 (37.3)^∗∗∗^

Abbreviations: mMNSI: modified Michigan Neuropathy Screening Instrument; DSPN: distal symmetrical polyneuropathy; DPNP: diabetic peripheral neuropathic pain; NCS: nerve conduction studies; DN4: Douleur Neuropathique; F: females; M: males; y: years; BMI: body mass index; T2DM: type II diabetes mellitus; HTN: hypertension; HbA1C: glycated haemoglobin; Vit. B_12_: vitamin B_12_; HTN: hypertension; Hom: homocysteine; T-CHOL: total cholesterol; HDL-C: high-density lipoprotein cholesterol; LDL-C: low-density lipoprotein cholesterol; TG: triglyceride; eGFR: estimated glomerular filtration rate. ^a^Symptomatic DSPN was defined as complaint of symmetrical, bilateral, distal sensory and/or motor defects. Data were presented as *n* of F/M for gender, *n* (%) for categorical variables, and mean ± standard deviations (SD) for continuous values by the symptomatic and asymptomatic patients. Data were compared using the chi-squared test for categorical variables and the two-sample *t*-test for continuous ones. ^∗^*p* < 0.05, ^∗∗^*p* < 0.01, and ^∗∗∗^*p* < 0.0001 indicate significant difference when comparing the symptomatic and asymptomatic patients.

## Data Availability

Readers can access the data supporting the conclusions of the study via contact with the corresponding author.

## Supplementary Materials

Supplementary Materials Table S1: modified Michigan Neuropathy Screening Instrument was used for an evaluation of diabetic neuropathy. This table is composed of foot appearance, ulceration, ankle reflex, thermal threshold test, and vibration threshold test. The range of score was from 0 to 10. Table S2: the severity of distal symmetrical polyneuropathy accorded to the finding of nerve conduction studies. A severity is defined according to the number of abnormal data of nerve conduction studies in total five nerves including 3 motor nerves (ulnar, deep peroneal, and tibial) and 2 sensory nerves (sural and ulnar). Table S3: the data of nerve conduction studies of 5 nerves included amplitudes of sensory nerve action potentials to distal stimulation and nerve conduction velocities of 2 sensory nerves, distal latencies and amplitudes of compound muscle action potentials, and nerve conduction velocities and latencies of F wave for 3 motor nerves. All these parameters showed statistical significance between distal symmetrical polyneuropathy (DSPN)/diabetic peripheral neuropathic pain and no DSPN subjects. Table S4: the occurrence of focal median neuropathy or carpal tunnel syndrome was shown in the different distal symmetrical polyneuropathy (DSPN) and no DSPN groups. Focal median neuropathies are common in diabetic patients. Table S5: according to performance of the modified Michigan Neuropathy Screening Instrument (mMNSI), patients with mMNSI > 2.5 and abnormal vibration or thermal test results are likely to have DSPN (minimal DSPN); patients with MNSI > 2.5 but with normal vibration and thermal test results are likely to have no DSPN.
