# Supplementary material for: Diabetic Distal Symmetrical Polyneuropathy: Correlation of Clinical, Laboratory, and Electrophysiologic Studies in Patients with Type 2 Diabetes Mellitus
Source: J Diabetes Res. 2020 Jul 3;2020:6356459. doi: 10.1155/2020/6356459 (PMC7362296; doi:10.1155/2020/6356459)
Supplement: Supplementary Materials — Table S1: modified Michigan Neuropathy Screening Instrument was used for an evaluation of diabetic neuropathy. This table is composed of foot appearance, ulceration, ankle reflex, thermal threshold test, and vibration threshold test. The range of score was from 0 to 10. Table S2: the severity of distal symmetrical polyneuropathy accorded to the finding of nerve conduction studies. A severity is defined according to the number of abnormal data of nerve conduction studies in total five nerves including 3 motor nerves (ulnar, deep peroneal, and tibial) and 2 sensory nerves (sural and ulnar). Table S3: the data of nerve conduction studies of 5 nerves included amplitudes of sensory nerve action potentials to distal stimulation and nerve conduction velocities of 2 sensory nerves, distal latencies and amplitudes of compound muscle action potentials, and nerve conduction velocities and latencies of F wave for 3 motor nerves. All these parameters showed statistical significance between distal symmetrical polyneuropathy (DSPN)/diabetic peripheral neuropathic pain and no DSPN subjects. Table S4: the occurrence of focal median neuropathy or carpal tunnel syndrome was shown in the different distal symmetrical polyneuropathy (DSPN) and no DSPN groups. Focal median neuropathies are common in diabetic patients. Table S5: according to performance of the modified Michigan Neuropathy Screening Instrument (mMNSI), patients with mMNSI > 2.5 and abnormal vibration or thermal test results are likely to have DSPN (minimal DSPN); patients with MNSI > 2.5 but with normal vibration and thermal test results are likely to have no DSPN. [file 6356459.f1.docx]

**Table S1. Modified physical assessment of Michigan Neuropathy Screening Instrument (MNSI).**

| 1. | Appearance of Feet | | | | | | | | | | | | | | | | | | |
| --- | --- | --- | --- | --- | --- | --- | --- | --- | --- | --- | --- | --- | --- | --- | --- | --- | --- | --- | --- |
|  | **Right** | | | | | | | |  | | **Left** | | | | | | | | |
|  | a. | Normal | | □0 Yes | | □1 No | | |  | | a. | | Normal | | □0 Yes | | | □1 No | |
|  | b. | If no, check all that apply: | | | | | | |  | | b. | | If no, check all that apply: | | | | | | |
|  |  | Deformities  Dry skin, callus  Infection  Fissure  Other | | | | □  □  □  □  □ | | |  | |  | | Deformities  Dry skin, callus  Infection  Fissure  Other | | | | | □  □  □  □  □ | |
|  |  | specify: | | | | | | |  | |  | | specify: | | | | | | |
|  |  |  | | | | | | |  | |  | |  | | | | | | |
|  |  | | **Right** | | | | | | | |  | **Left** | | | | | | | |
| 2. | Ulceration | | Absent | | | | | Present | | |  | Absent | | | | | Present | | |
|  |  | | □ 0 | | | | | □ 1 | | |  | □ 0 | | | | | □ 1 | | |
| 3. | Ankle Reflexes | | Present | | Present/ Reinforcement | | | | | Absent |  | Present | | Present/ Reinforcement | | | | | Absent |
|  |  | | □ 0 | | □ 0.5 | | | | | □ 1 |  | □ 0 | | □ 0.5 | | | | | □ 1 |
| 4. | Vibration threshold test* | | Present | | | | Abnormal | | | |  | Present | | | | Abnormal | | | |
|  |  |  | □ 0 | | | | □ 1 | | | |  | □ 0 | | | | □ 1 | | | |
| 5 | Thermal | | Normal | | | | Abnormal | | | |  | Normal | | | | Abnormal | | | |
|  | threshold test^#^ | | □ 0 | | | | □ 1 | | | |  | □ 0 | | | | □ 1 | | | |
| Total Score /10 Points | | | | | | | | | | | | | | | | | | | |

^*^: quantitative vibration threshold of big toe pulp, using vibratory stimulator of VSA-3000 devices, Medoc Ltd. Advanced Medical Systems, Ramat Yishai, Israel.

^#^: quantitative thermal threshold of dorsal lateral foot, thermode of Medoc Thermal Sensory Analyzer (TSA)-2001 devices, Medoc Ltd. Advanced Medical Systems, Ramat Yishai, Israel.

MNSI, ^©^ University of Michigan, 2000.

**Table S2. The severity score of neuropathy for the nerve conduction study.**

| Nerve (Reference of NCV, mean±2SD, m/s) [reference of amplitude] |  | Nerve Conduction Velocities | | | |  | Points | Findings |
| --- | --- | --- | --- | --- | --- | --- | --- | --- |
|  |  | Right | |  | Left |  |  |  |
| Motor |  |  | |  |  |  | (0 or 1) | (N or A) |
| Tibial (50±6.8)  Peroneal (51.1±6.4)  Ulnar (60.5±8.4) |  | |  |  |  |  |  |  |
|  |  | |  |  |  |  |  |  |
|  |  | |  |  |  |  |  |  |
| Sensory |  |  | |  |  |  |  |  |
| Ulnar (66±10.2) |  |  | |  |  |  |  |  |
| Sural (49.4±8.4) or [< 10 µV] |  |  | |  |  |  |  |  |
| N or 0: Normal A or 1: Abnormal in one limb at least | | | | | | | | |
| **Total Score：_________**  (Severity of Neuropathy 0-1: normal, 2: mild, 3-4: moderate, 5: severe) | | | | | | | | |

Abbreviations: NCV, nerve conduction velocity; SD: standard deviation.

**Table S3. Distal latencies of compound muscle action potentials and F wave of nerve conduction studies by DSPN stage and DPNP status.**

|  |  |  |  |  | DSPN without DPNP | | | |  |
| --- | --- | --- | --- | --- | --- | --- | --- | --- | --- |
|  | No DSPN and no DPNP | No  DSPN | Minimal DSPN | Subclinical DSPN without DPNP | All  DSPN | Mild DSPN | Moderate DSPN | Severe DSPN | DPNP |
| *Patient No.* | *59* | *64* | *19* | *17* | *34* | *15* | *9* | *10* | *16* |
| Sensory |  |  |  |  |  |  |  |  |  |
| Ulnar nerve |  |  |  |  |  |  |  |  |  |
| Amp, µV | 32.8±13.8 | 32.5±13.6 | 28.5±13.6 | 23.1±14.1^*^ | 13.6±10.1^***,†††^ | 20.3±9.9 | 10.0±6.6 | 6.9±6.6 | 18.7±13.2^***^ |
| NCV, m/s | 56.1±5.1 | 56.3±5.1 | 54.6±3.8 | 48.5±4.7^***^ | 43.6±17.2^***,††^ | 52.9±5.7 | 43.6±16.5 | 29.6±20.7 | 46.7±19.2^**^ |
| Sural nerve |  |  |  |  |  |  |  |  |  |
| Amp, µV | 14.6±7.0 | 14.6±7.1 | 12.6±3.1 | 10.5±6.1^*^ | 5.0±4.3^***,††^ | 7.0±4.3 | 5.4±3.6 | 1.5±2.5 | 8.1±7.2^**^ |
| NCV, m/s | 52.9±7.9 | 52.1±10.2 | 52.6±8.9 | 43.5±13.1^***^ | 33.2±23.1^***,††^ | 45.9±14.7 | 33.8±21.7 | 13.7±22.2 | 37.4±19.9^***^ |
| Motor |  |  |  |  |  |  |  |  |  |
| Ulnar nerve |  |  |  |  |  |  |  |  |  |
| Distal latencies (ms) | 2.6±0.3 | 2.6±0.3 | 2.7±0.3 | 3.0±0.3^***^ | 3.1±0.5^***,†††^ | 2.8±0.2 | 3.1±0.5 | 3.6±0.6 | 3.3±1.5^***^ |
| Amp, mV | 10.0±2.0 | 9.9±1.9 | 10.4±2.1 | 9.1±1.9 | 8.3±2.8^**^ | 9.2±1.9 | 8.0±2.9 | 7.1±3.4 | 7.7±3.1^***^ |
| NCV, m/s | 57.4±3.7 | 57.2±3.8 | 56.8±5.2 | 50.9±6.0^***^ | 50.1±5.5^***,†††^ | 54.3±3.3 | 49.8±3.5 | 44.0±3.5 | 45.4±18.3^***^ |
| Peroneal nerve |  |  |  |  |  |  |  |  |  |
| Distal latencies (ms) | 3.6±0.7 | 3.6±0.7 | 3.6±0.4 | 4.3±1.3^*^ | 4.4±0.9^***^ | 4.1±0.8 | 4.8±0.8 | 4.9±1.1 | 4.3±1.1^*^ |
| Amp, mV | 4.5±2.1 | 4.4± 2.1 | 4.6±2.2 | 2.4±1.9^***^ | 2.5±1.9^***,†^ | 3.3±1.8 | 2.3±1.3 | 1.5±2.0 | 2.9±2.1^**^ |
| NCV, m/s | 45.6±4.1 | 45.6± 4.1 | 45.7±3.1 | 37.9±4.0^***^ | 34.6±11.6^***,††^ | 40.3±2.4 | 36.5±3.0 | 24.5±17.1 | 37.8±11.7^***^ |
| Tibial nerve |  |  |  |  |  |  |  |  |  |
| Distal latencies (ms) | 3.7±0.5 | 3.7±0.5 | 3.8±0.5 | 4.6±1.0^***^ | 4.3±0.9^***,†^ | 4.0±0.9 | 4.4±0.5 | 4.9±0.9 | 4.1±0.6^**^ |
| Amp, mV | 12.9±3.9 | 12.7± 4.0 | 12.9±5.2 | 12.5±4.6 | 8.5±4.8^***,†^ | 10.3±5.2 | 9.0±2.4 | 5.4±4.5 | 8.0±5.0^***^ |
| NCV, m/s | 44.9±3.6 | 45.0± 3.5 | 42.9±2.4^*^ | 39.1±3.8^***^ | 37.7±4.8^***,†††^ | 40.7±4.2 | 37.3±1.7 | 33.4±4.1 | 38.7±12.5^**^ |
| F-wave |  |  |  |  |  |  |  |  |  |
| Ulnar nerve, Latencies (ms) |  |  |  |  |  |  |  |  |  |
| n | 59 | 64 | 19 | 17 | 34 | 15 | 9 | 10 | 14 |
| Latencies (ms) | 25.7±2.2 | 25.7±2.2 | 26.7±2.0 | 28.9±2.0^***^ | 30.0±3.0^***,†††^ | 27.9±2.1 | 30.8±3.3 | 32.5±1.6 | 29.5±3.9^***^ |
| Peroneal nerve, Latencies (ms) |  |  |  |  |  |  |  |  |  |
| n | 59 | 64 | 18 | 14 | 26 | 14 | 8 | 4 | 13 |
| Latencies (ms) | 46.0±3.9 | 46.0±4.0 | 48.0±3.8 | 51.1±4.4^***^ | 52.6±3.5^***,†^ | 51.2±2.8 | 53.0±2.3 | 56.7±4.5 | 53.5±11.0^***^ |
| Tibial nerve, Latencies (ms) |  |  |  |  |  |  |  |  |  |
| n | 59 | 64 | 19 | 17 | 33 | 15 | 9 | 9 | 14 |
| Latencies (ms) | 46.0±3.8 | 46.0±3.9 | 47.6±4.2 | 50.9±3.5^***^ | 53.9±5.8^***,††^ | 50.4±4.6 | 55.2±4.2 | 58.4±5.6 | 51.0±5.7^***^ |

Abbreviations: mMNSI, modified Michigan Neuropathy Screening Instrument; DSPN, distal symmetrical polyneuropathy; DPNP, diabetic peripheral neuropathic pain; NCS, nerve conduction studies; DN4, Douleur Neuropathique; DL, distal latency; n, the numbers that presence of compound muscle action potentials or F waves.

^a^ DPNP indicates patients with DN4≧4.

^b^ No DSPN, patients with mMNSI total score <2.5 and NCS severity score 0-1; minimal DSPN, patients with mMNSI total score ≧2.5 and NCS severity score 0-1; subclinical DSPN, patients with mMNSI total score <2.5 and NCS severity score ≧2; DSPN, patients with mMNSI total score≧2.5 and NCS severity score ≧2

Data were presented as mean ± standard deviations (SD) for continuous values, given DSPN stage and with/without DPNP.

Data were compared with patients with either no DSPN or no DPNP using two sample t-test; and data were also compared by DSPN severity (mild, moderate, severe) via one-way ANOVA test.

^*^P<0.05, ^**^p<0.01, ^***^p<0.0001 indicates significantly different in comparison with patients with either no DSPN or no DPNP.

^†^P<0.05, ^††^p<0.01, ^†††^p<0.0001 indicates significantly different by DSPN severity.

**Table S4. Focal median neuropathy at wrist or carpal tunnel syndrome by DSPN stage**^a^**.**

|  |  |  |  | DSPN | | | |
| --- | --- | --- | --- | --- | --- | --- | --- |
|  | No DSPN | Minimal DSPN | Subclinical DSPN | All DSPN | Mild DSPN | Moderate DSPN | Severe DSPN |
| *Patient No.* | *64* | *19* | *18* | *44* | *18* | *14* | *12* |
| Focal median neuropathy at wrist^b^, n(%) | 30 (46.9) | 8 (42.1) | 13 (72.2) | 30 (68.2) | 11 (61.1) | 12 (85.7) | 7 (58.3) |
| CTS^c^, n(%) | 9 (30.0) | 2 (25.0) | 3 (23.1) | 3 (10.0) | 1 (9.1) | 2 (16.7) | 1 (14.3) |

Abbreviations: mMNSI, modified Michigan Neuropathy Screening Instrument; DSPN, distal symmetrical polyneuropathy; CTS, Carpal tunnel syndrome; NCS, nerve conduction studies.

^a^ No DSPN, patients with mMNSI total score <2.5 and NCS severity score 0-1; minimal DSPN, patients with mMNSI total score ≧2.5 and NCS severity score 0-1; subclinical DSPN, patients with mMNSI total score <2.5 and NCS severity score ≧2; DSPN, patients with mMNSI total score≧2.5 and NCS severity score ≧2

^b^ according to nerve conduction studies.

^c^ clinical evidence of carpal tunnel syndrome before the nerve conduction studies.

**Table S5. Performance of the modified MNSI.**

|  | DSPN stage | | | | |
| --- | --- | --- | --- | --- | --- |
|  | No DSPN | Minimal DSPN | Subclinical DSPN | DSPN | DPNP |
| Patients grouped according to modified MNSI (with MNSI >2.5) | 64 | 19 | 18 | 44 | 16 |
| Patients grouped according to modified MNSI (with MNSI >2.0) | 34 | 49 | 6 | 56 | 16 |
| Patients grouped according to modified MNSI (with MNSI >2.5) given either with abnormal vibration or thermal test | 6 | 19 | 2 | 44 | 10 |

Results were summarized as number of patients.

Abbreviations: MNSI, Michigan Neuropathy Screening Instrument; DPNP, diabetic peripheral neuropathic pain; DSPN, distal symmetrical polyneuropathy.
